# Histopathological grading systems analysis of oral squamous cell carcinomas of young patients

**DOI:** 10.4317/medoral.20953

**Published:** 2016-03-06

**Authors:** Juliana-Cristina Frare, Iris Sawazaki-Calone, Ana-Lucia-Carrinho Ayroza-Rangel, Alexandre-Galvão Bueno, Carlos-Floriano de Morais, Hildebrando-Massahiro Nagai, Reno Kunz, Marcio-Ajudarte Lopes

**Affiliations:** 1 Western Parana State University, Cascavel, PR, Brazil; 2 Oral Pathology and Oral Medicine, Dentistry School, Western Parana State University, Cascavel, PR, Brazil; 3 ANATOM Anatomic Pathology to Laboratoy, Cascavel, PR, Brazil; 4 APC Anatomic Pathology Laboratory, Cascavel, PR, Brazil; 5 UOPECCAN Cancer Hospital, Cascavel, PR, Brazil; 6 Oncology Center of Cascavel (CEONC), Cascavel, PR, Brazil; 7 Oral Diagnosis Department, Piracicaba Dental School, University of Campinas, UNICAMP, SP, Brazil

## Abstract

**Background:**

To analyze the clinicopathological profile of young patients (≤ 40 years) with oral SCC and correlate with a control group (≥ 50 years) by means of histopathological grading systems.

**Material and Methods:**

14 young patients and 14 control patients were selected with similar clinical stage and tumor location. Demographic and clinical data were obtained from patient records and histological sections were evaluated according to four histopathological grading systems. Associations between categories of demographic and clinical data were performed through Chi-square test and Exact Fisher test. The survival analyzes were performed according to the Kaplan-Meier method.

**Results:**

The comparison between groups showed a greater association of treatment modalities in younger patients (*p*=0.022), they had a higher incidence of local recurrence and regional metastasis (*p*=0.018) and lower disease-free survival in 5 years (*p*=0.069). There was no difference in 5-year overall survival among the studied groups. There was no difference in histological grading between studied groups according to the four used systems.

**Conclusions:**

This study showed that, despite tumors had similar histological grade and more therapeutic modalities were used in the young group, tumors in young patients had a higher incidence of recurrence/metastasis, showing tendency to a more aggressive behavior.

**Key words:**Squamous cell carcinoma, tumors histological grading, young.

## Introduction

Squamous cell carcinoma (SCC) originates in the stratified squamous epithelium and represents 90-95% of all malignant neoplasms in the oral cavity (1). This disease affects mostly males, after the fifth decade of life and is strongly associated with alcohol and tobacco abuse (2).

The oral SCC is an uncommon disease in patients under the age of 40 years old, and its incidence ranges from 0.4 to 6% of the cases. However, in recent years this number has been increasing gradually (3-5). According to some authors, young patients with SCC have a distinct clinical profile and limited association with traditional risk factors (1,3,5,6). Furthermore, it is considered that the process of oncogenesis in young adults may be different from that which occurs in elderly patients (7,8). According to Santos-Silva *et al.* (5), the high incidence of abnormalities in cellular DNA suggests that young patients with oral cancer may have increased genomic instability, indicating genetic differences between the disease of these patients and the elderly.

In order to standardize information, it was developed the “TNM Classification of Malignant Tumors System”, which, today, is still the most used clinical staging system. However, this system has some limitations, especially in relation to previewing prognosis, as some patients with early oral SCC evolve badly and others with advanced tumors survive (9). It is considered that its greatest disadvantage is the inability to follow the advances in the understanding of cancer biology and incorporate new prognostic variables, as they become available (10).

In order to fill this gap, histopathological classifications for oral SCC have been developed in order to explain the divergent biological behavior of tumors with apparently similar clinical features. Many authors, at different times, proposed new histological grading systems for tumors in an attempt to predict their clinical behaviour (11-14).

Based on these data, this study aimed to test the hypothesis if comparison the outcomes of similar clinical stages between younger patients (≤ 40 years) and older patients (patients ≥ 50 years), the aggressive nature of squamous cell carcinoma in younger individuals is due to a higher pathologic grade of the tumor.

## Material and Methods

All patients aged under 40 years old with primary intra-oral SCC treated at the Parana Western Union Hospital for Studies and Cancer Combat - Uopeccan and Cascavel Oncology Center - Ceonc from 1998 to 2013 were retrieved.

Inclusion criteria were records with complete clinicopathological and demographic data, treatment based on surgery with or without adjuvant radiotherapy and/or chemotherapy and viability for analysis of tumor tissue embedded in paraffin blocks. The Research Ethics Committee of the Piracicaba Dental School, State University of Campinas, Protocol 100/2012 approved this study.

The demographic data (age and gender), social habits (tobacco and alcohol consumption), tumor location, TNM stage, surgical margins, lymph node involvement, recurrence, metastasis, treatment and the patient’s current status were obtained from medical records. The results were compared with a control group (age ≥ 50 years) selected in a paired form of treated patients files in the period in the same institutions and with similar clinicopathological features ( Table 1, Table 2).

**Table 1:**
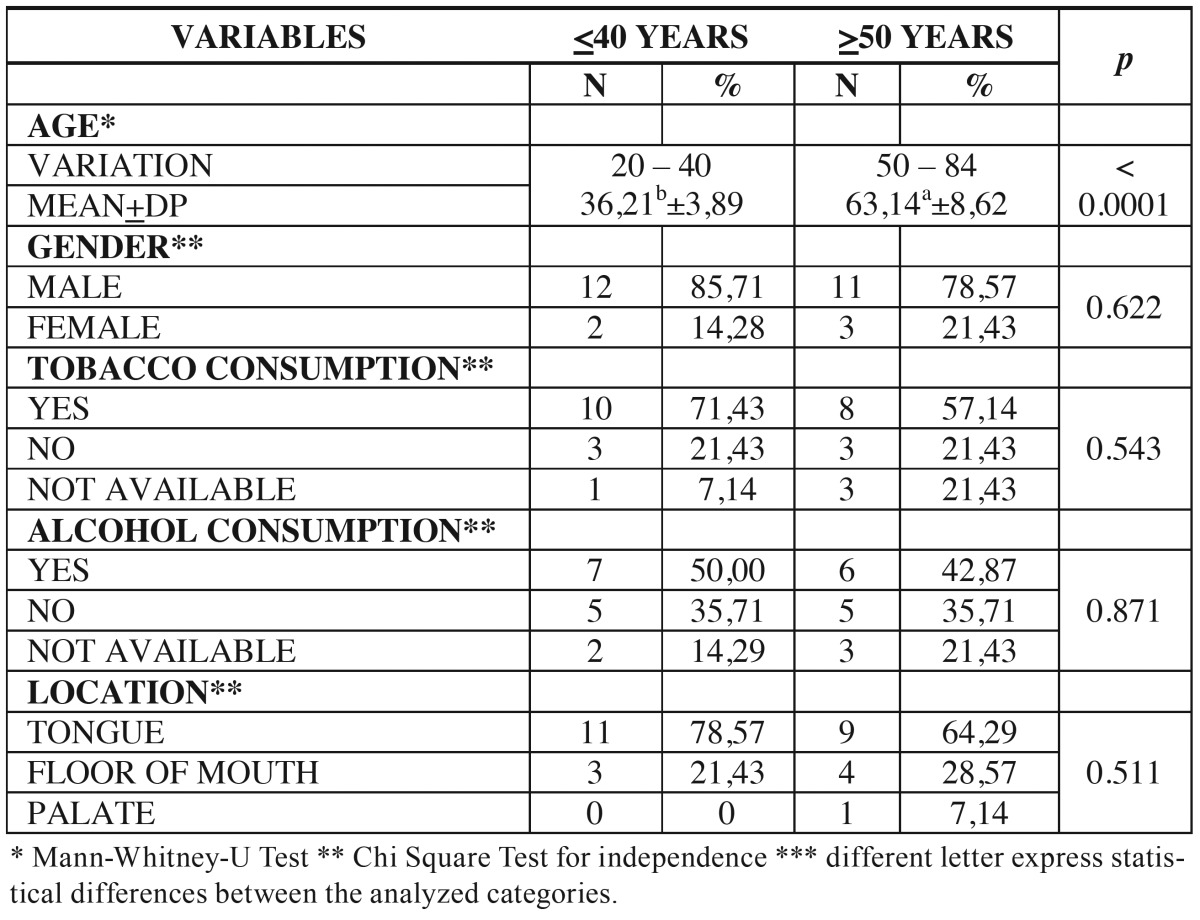
Distribution of patients according to age, gender, habits and location of the tumor.

**Table 2:**
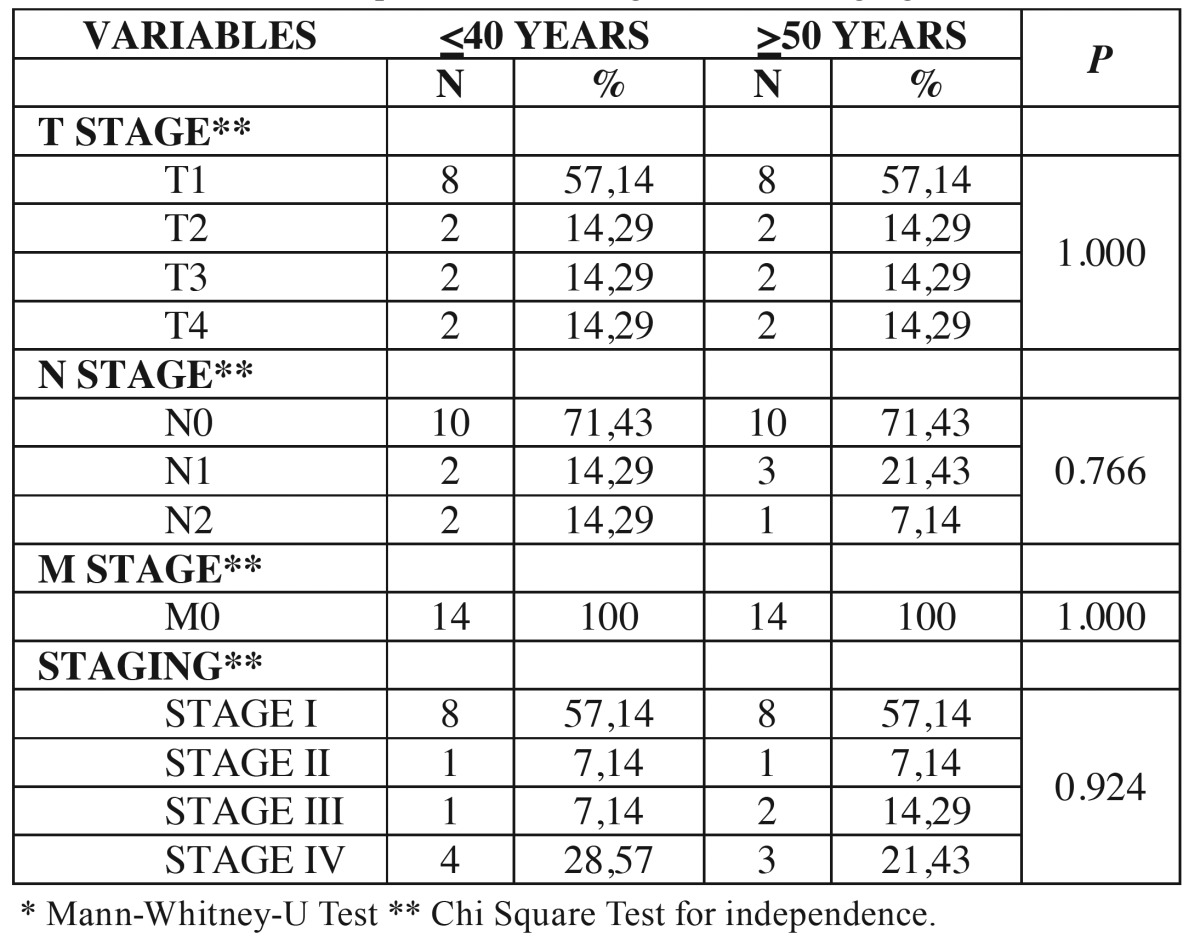
Distribution of patients according to clinical staging.

- Histopathological analysis 

After the sample selection, new histological sections with 4 µm thick were obtained from the paraffin blocks corresponding to surgical specimens and stained with hematoxylin and eosin (HE). The slides were evaluated using an optical microscope according to four histopathologic grading systems: 1) World Health Organization System - WHO System, 2) Malignancy Invasive Margins Deep Grading System - MG system (12), 3) Histological Risk Model - HR System (13) and 4) BD Risk Score (14).

Scoring was carried out simultaneously by two calibrated authors (ISC and ALCAR) until consensus was achieved. To determine the intra-observer degree of agreement, 30% (n = 34) of the samples were randomly selected and examined twice. The intra-observer Cohen’s kappa coefficients were 0.83 for WHO grading system, 0.86 for MG system, 0.84 for HR model, and 0.88 for BD model. All investigators were blinded to demographic and clinical data and outcomes.

- Statistical analysis

The associations between the categories of demographic and clinical data, as well as diagnostic of histopathological grading systems of the tumors were performed using the Chi-square test for independence and Fisher exact test. The age data was evaluated for standard distribution using the Shapiro-Wilk normality test and homogeneity of variance by F test. As these assumptions were not accepted, the two age groups were compared using the non parametric Mann-Whitney. The analysis of overall survival and disease-free survival were performed according to the Kaplan-Meier method, comparing the two age classes through the Gehan’s Wilcoxon test. The significance level was 0.05. Analyses were performed in the statistical package Statistica 7.0 (Statsoft, 2004).

## Results

During the studied period, were identified 22 patients aged under 40 years with oral SCC in the institutions surveyed. Of these 22 patients, 14 (63.64%) met the inclusion criteria. The mean age of these 14 patients was 36.21 years (± 3.89), ranging from 20 to 40 years. Most patients were male 12 (85.71%) and 2 (14.29%) were female. Regarding the social habits, 10 (71.43%) reported smoking and 7 (50%) alcohol consumption. The mean age of the control group patients was 63.14 years (± 8.62), ranging from 50 to 84 years. Most were male 11 (78.57%) and 3 (21.43%) were female. According to the habits, 8 (57.14%) reported tobacco and 6 (42.87%) alcohol consumption. Regarding the tumor location, 11 (78.57%) developed on the tongue and 3 (21.43%) on the floor of mouth in the group of young patients. In the control group, 9 (64.29%) occurred on the tongue, 4 (28.57%) on the floor of mouth and 1 (7.14%) on the palate. The comparison between groups of young patients and control patients showed no significant differences regarding gender (*p* = 0.622), smoking habit (*p* = 0.543), alcohol consumption (*p* = 0.871) and tumor location (*p* = 0.511) ( Table 1).

In both groups, young and control, 10 patients (71.42%) were classified as early stages T1-T2 and 4 (28.58%) as advanced stage T3-T4. Regarding the stage N, 10 patients (71.73%) in each group had non-metastatic regional lymph nodes (N0). In the youth group, 2 patients (14.29%) were N1 and 2 (14.29%) were N2. In the control group, 3 patients (21.43%) were N1 and 1 patient (7.14%) was N2. For distant metastasis, in both groups all 14 patients (100%) were M0. Clinical staging in both groups showed that 9 patients (64.28%) were classified as stage I and II, and 5 patients (35.72%) as stages III and IV. The comparison between groups of young patients and control patients showed no difference in the T stage (*p* = 1.000), N stage (*p* = 0.766), M stage (*p* = 1.000) and clinical stage (*p* = 0.924) ( Table 2).

As for treatment performed in young patients, 4 (28.57%) underwent only surgery, 4 (28.57%) surgery associated with radiotherapy and 6 (42.86%) surgery associated with radiotherapy and chemotherapy. In control patients group, 7 (50%) were surgery and other 7 (50%) surgery associated with radiotherapy ( Table 3).

**Table 3:**
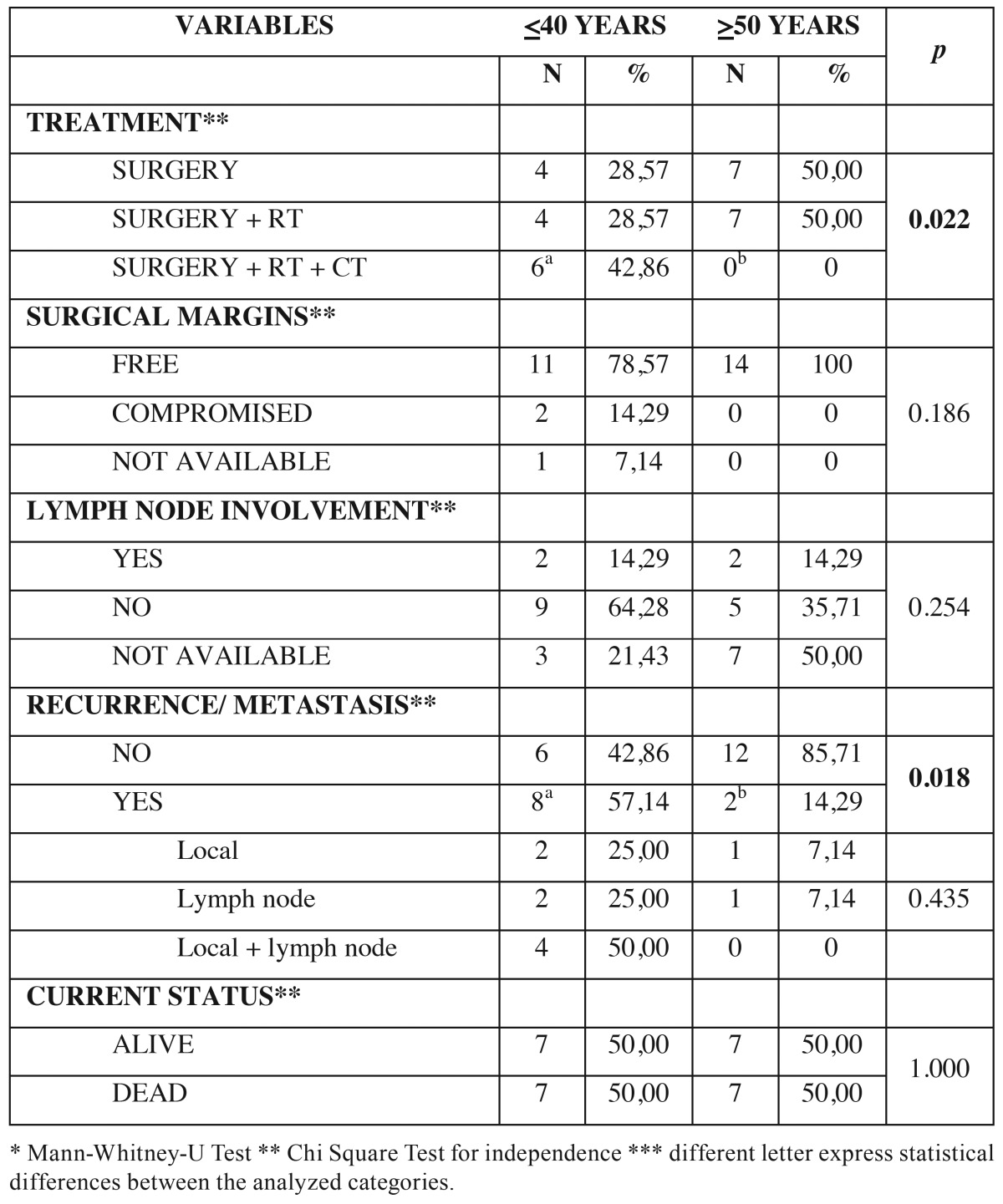
Patients’ distribution according to treatment and follow up.

The analysis of surgical margins showed that in 11 young patients (78.57%) the surgical margins were free and in 2 (14.29%) compromised. In one patient (7.14%) this information was not available. In all 14 control patients (100%) the surgical margins were free ( Table 3). Neck dissection was performed in 10 (71.42%) young patients and in 7 (50%) control patients. Histopathological confirmation of lymph node commitment was observed in 2 patients (14.29%) in each group ( Table 3).

In the clinical follow-up after cancer treatment, 8 young patients (57.14%) presented recurrence/metastasis compared to only 2 control patients (14.29%). Of the young patients with recurrence/metastasis, in 2 (25%) was local, in 2 (25%) was in lymph node and in 4 (50%) was local and in lymph node. In the control group, 1 patient (7.14%) had local recurrence and 1 (7.14%) in lymph node ( Table 3). Comparing both groups, young patients had almost 4 times more risk to develop recurrence/metastasis than the control group (OR=3.998). As for the current status of the patients, 7 (50%) in each group were alive and 7 (50%) dead. Of the dead patients, 5 (71.43%) in each group died due to tumor ( Table 3).

The comparison between young and control groups showed a greater association of treatment modalities used in younger patients (*p* = 0.022) and younger patients had higher rate of recurrence/metastasis (*p* = 0.018). On the other hand, regarding the surgical margins, lymph node commitment and current status did not differ between the groups, with *p* values respectively (*p* = 0.186) (*p* = 0.254) and (*p* = 1.000) ( Table 3).

**Figure 1:**
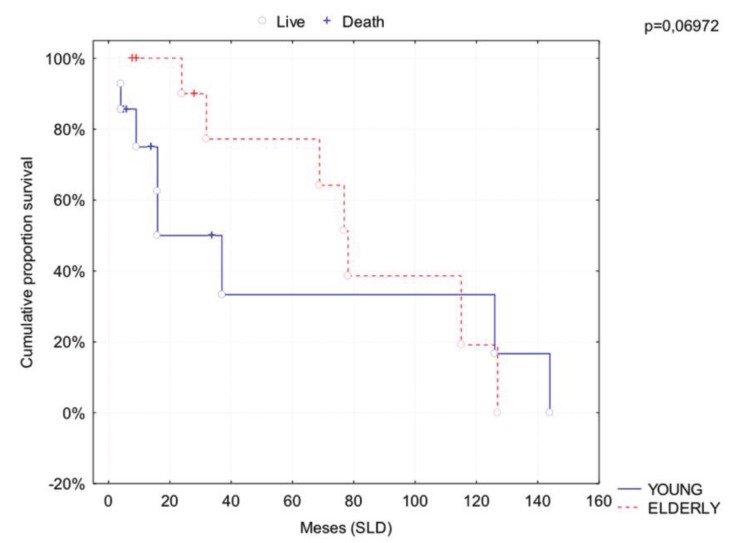
Comparative analysis of disease free survival rate among groups.

The overall survival rate (OS) in 5 and 10 years in the young group was 64% and 48%, respectively. In the control group, the overall survival rate (OS) in 5 and 10 years was 78% and 19%, respectively. Disease-free survival rate (DFS) for young patients was 37% in 5 years and 10 years. For the control patients the DFS was 78% in 5 years and 18% in 10 years. Comparing the two groups through Gehan’s Wilcoxon test, it was observed tendency to statistical difference in 5 years DFS (*p* = 0.069), where young patients had worse rate (Fig. 1). There was no significant difference in overall survival rate between the groups (*p* = 0.376).

The WHO grading system classified in both groups, young and control patients, 13 tumors (92.85%) as well or moderately differentiated and 1 (7.15%) as poorly differentiated. No significant associations were observed between the studied groups in the WHO grading system ( Table 4 and  Table 4 continue ).

**Table 4:**
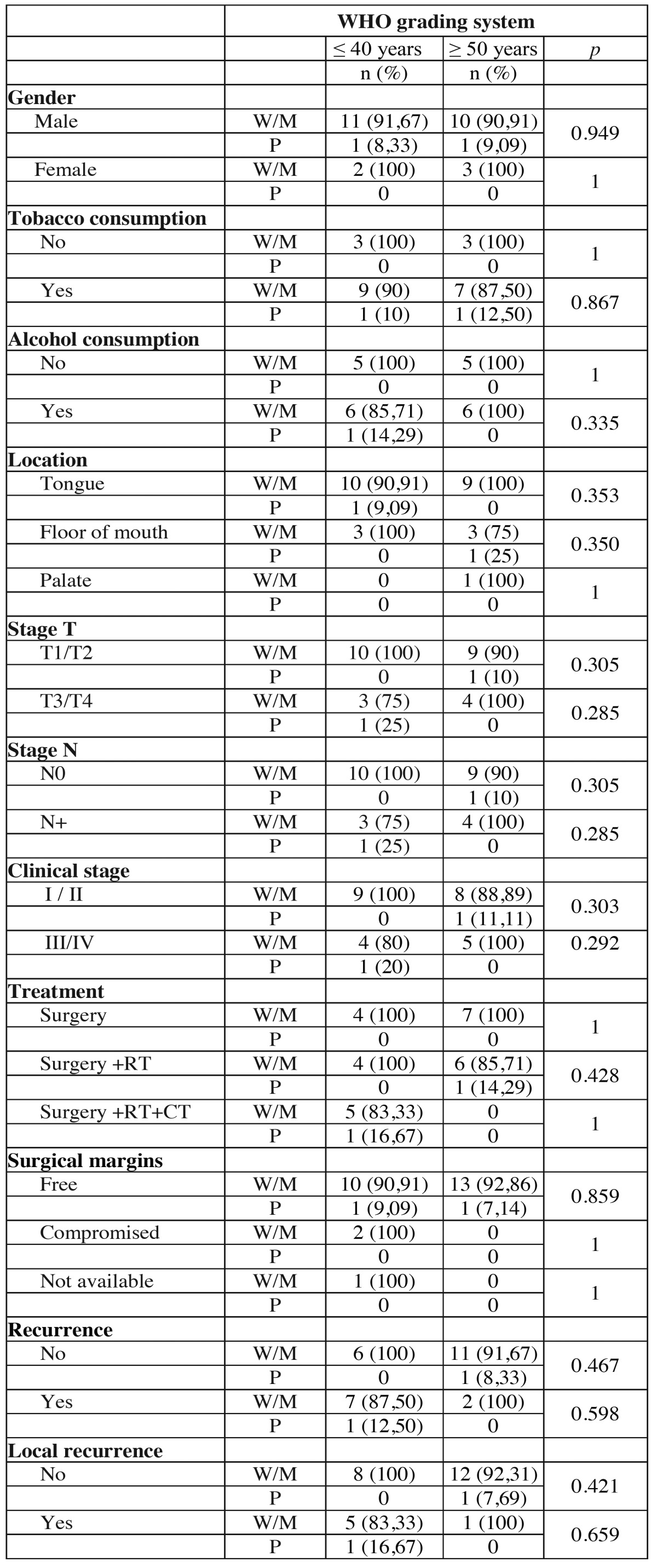
Association of clinical and demographic characteristics of the tumors of young patients (≤ 40 years) and control patients (≥ 50 years) with histopathological classification according to the WHO system.

**Table 4 Continue:**
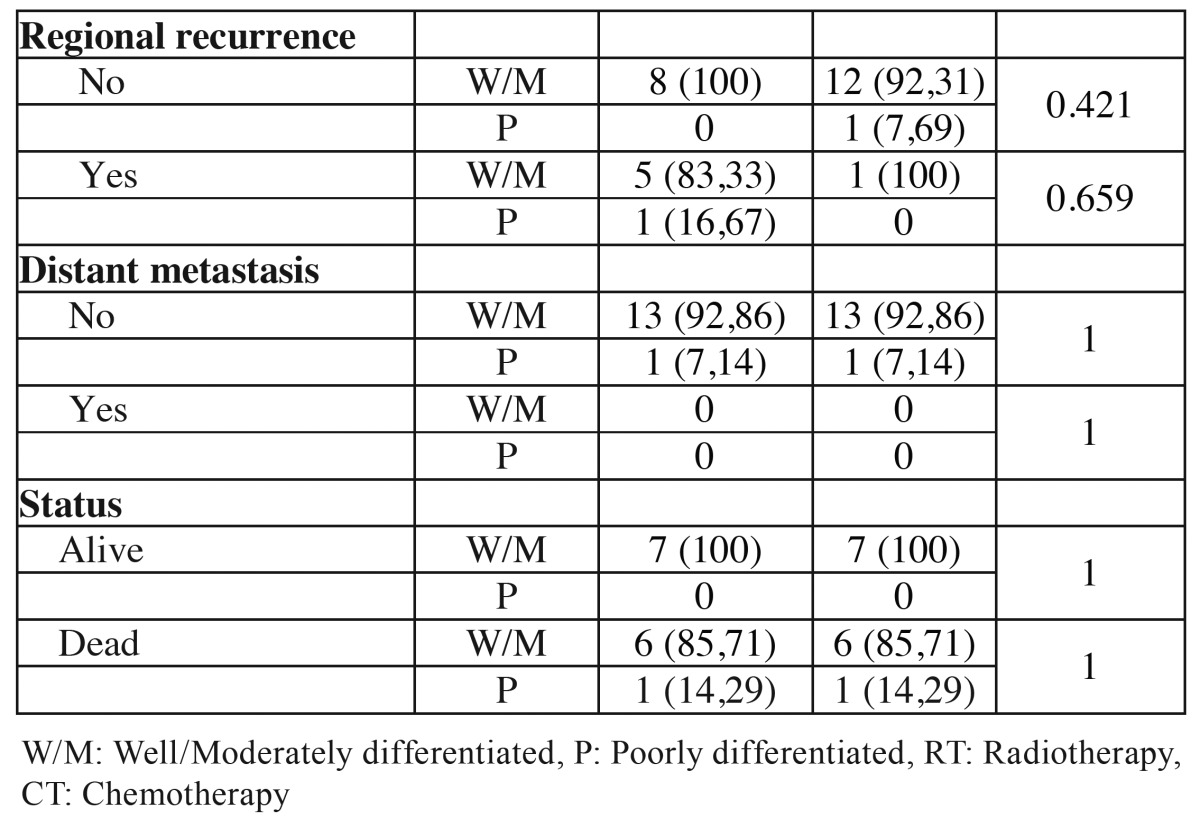
Association of clinical and demographic characteristics of the tumors of young patients (≤ 40 years) and control patients (≥ 50 years) with histopathological classification according to the WHO system.

The MG grading system ranked all 14 tumors of young and control patients as low or intermediate risk. In the young patients 1 tumor was classified as low risk and 13 (92.86%) as intermediate risk. In the control group 3 tumors were classified as low risk and 11 as intermediate risk. There were no significant associations with this classification in the comparative analysis between the groups ( Table 5 and  Table 5continue ).

**Table 5:**
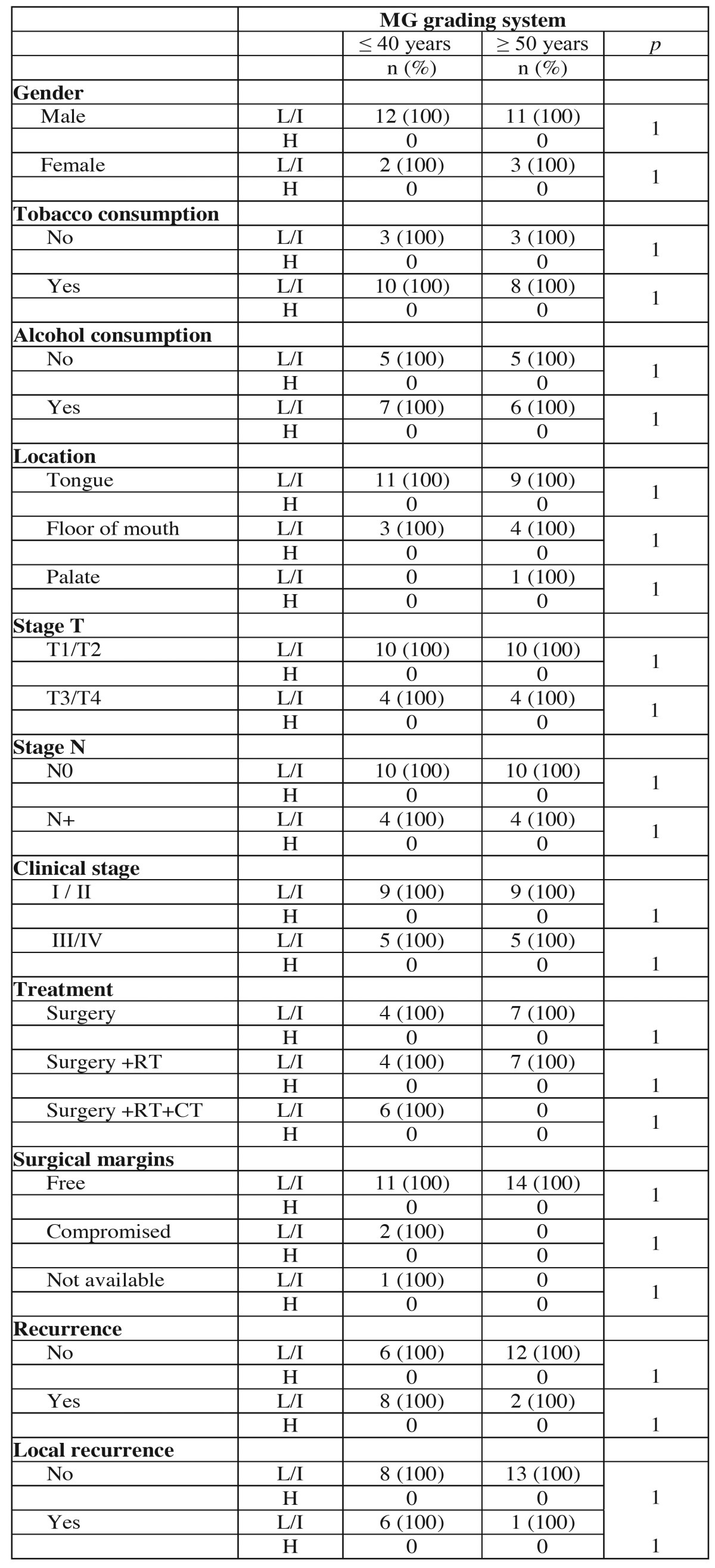
Association of clinical and demographic characteristics of the young patients (≤ 40 years) and control patients (≥ 50 years) with histopathological classification according to MG grading system.

**Table 5 Continue:**
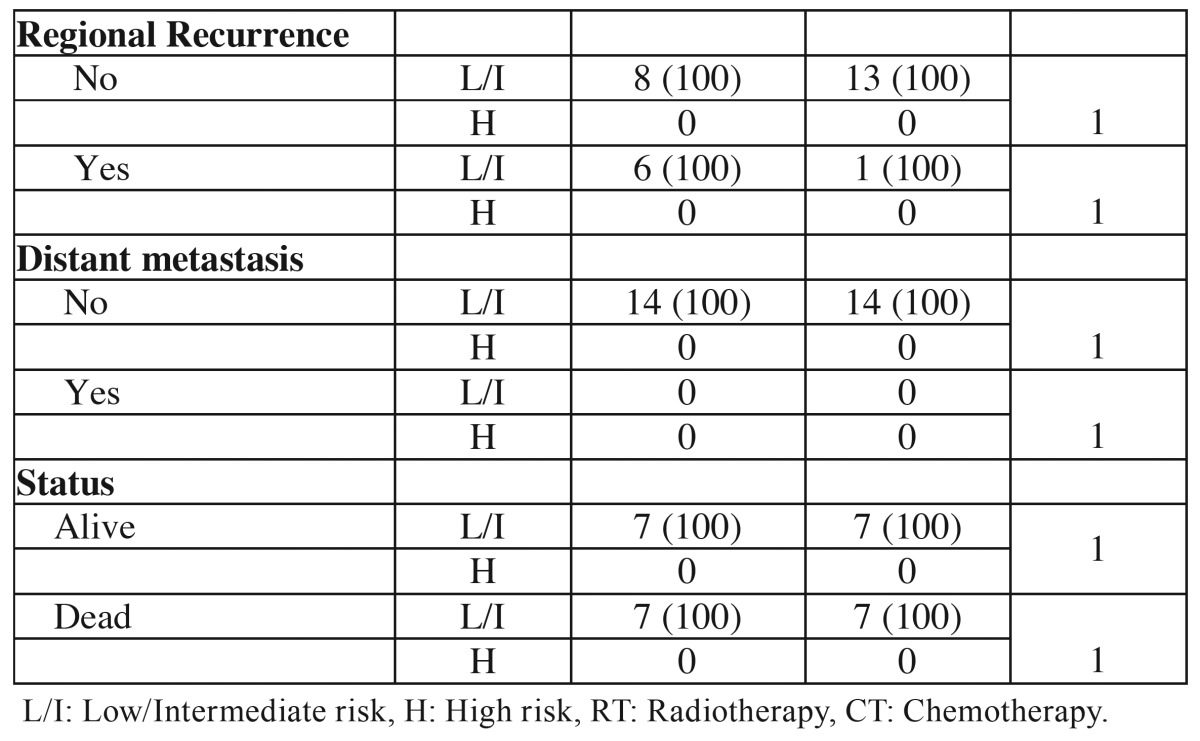
Association of clinical and demographic characteristics of the young patients (≤ 40 years) and control patients (≥ 50 years) with histopathological classification according to MG grading system.

In HR grading system, 7 tumors (50%) in the young group were classified as low or intermediate risk and 7 (50%) as high risk. In the control group 6 tumors (42.86%) were classified as low or intermediate risk and 8 (57.14) as high risk. When evaluating the relationship between the young and control groups, there were statistical differences in the variables clinical stage III/IV (*p* = 0.002), free surgical margins (*p* = 0.002) and absence of regional recurrence (*p* = 0.017) ( Table 6 and  Table 6 continue ).

**Table 6:**
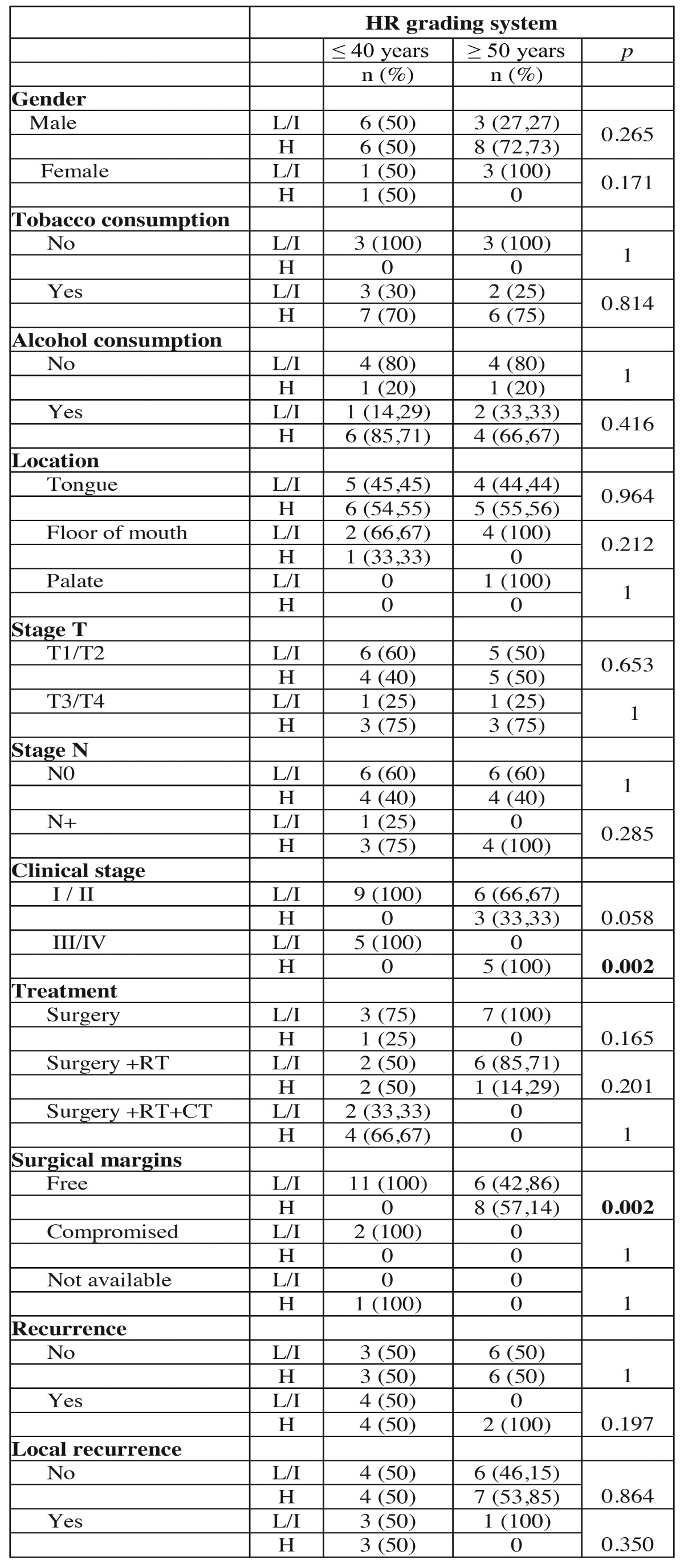
Association of clinical and demographic characteristics of the tumors of young patients (≤ 40 years) and control patients (≥ 50 years) with histopathological classification according to the HR grading system.

**Table 6 continue:**
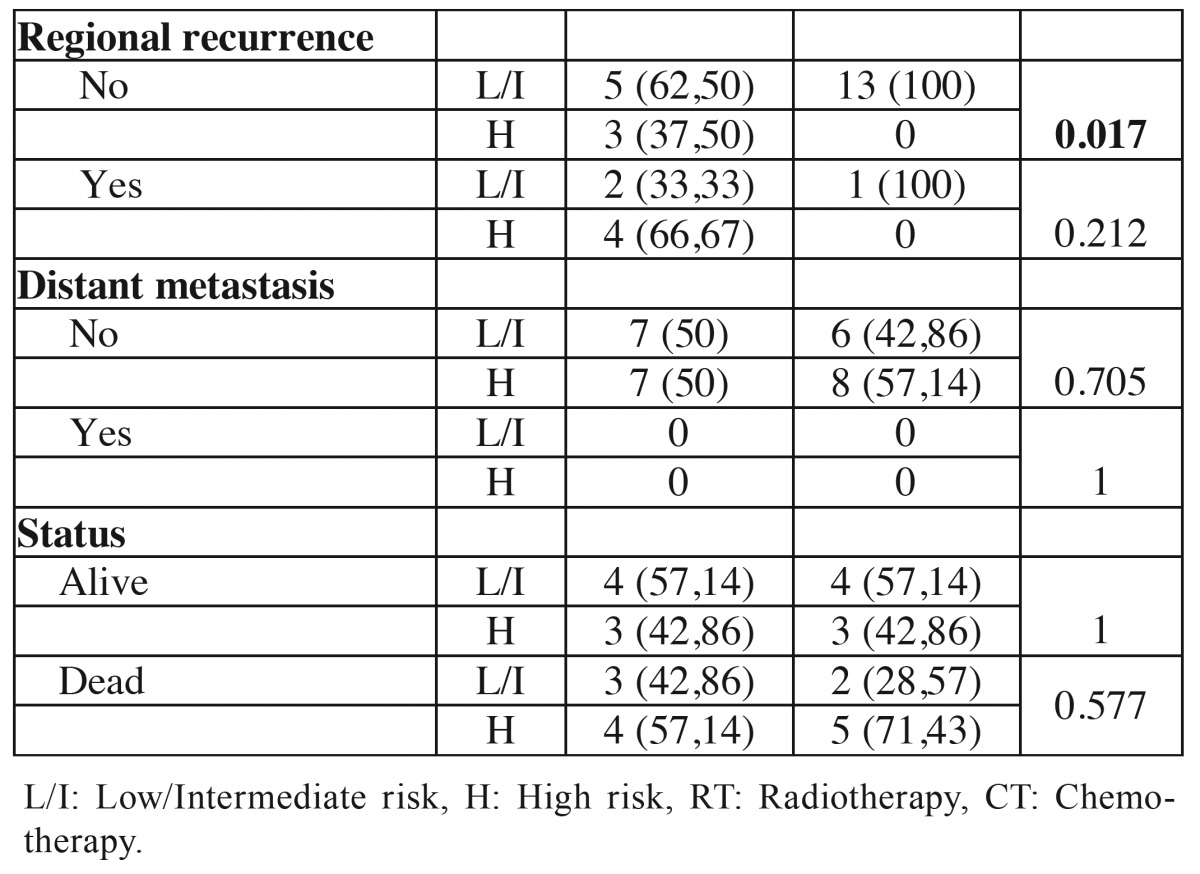
Association of clinical and demographic characteristics of the tumors of young patients (≤ 40 years) and control patients (≥ 50 years) with histopathological classification according to the HR grading system.

The BD risk score was performed in 13 young patients and in 6 was classified as low or intermediate risk and in 7 as high risk. In the control group 8 tumors as low or intermediate risk and in 6 as high risk. No significant correlation was observed between clinical parameters and the BD risk score in the comparison between groups ( Table 7 and  Table 7 Continue). Analyzing the results of the four grading system, it was noted that more tumors in both group ages were classified as poor differentiated or high risk in the HR and BD system comparing to WHO and MG systems ( Table 8).

**Table 7:**
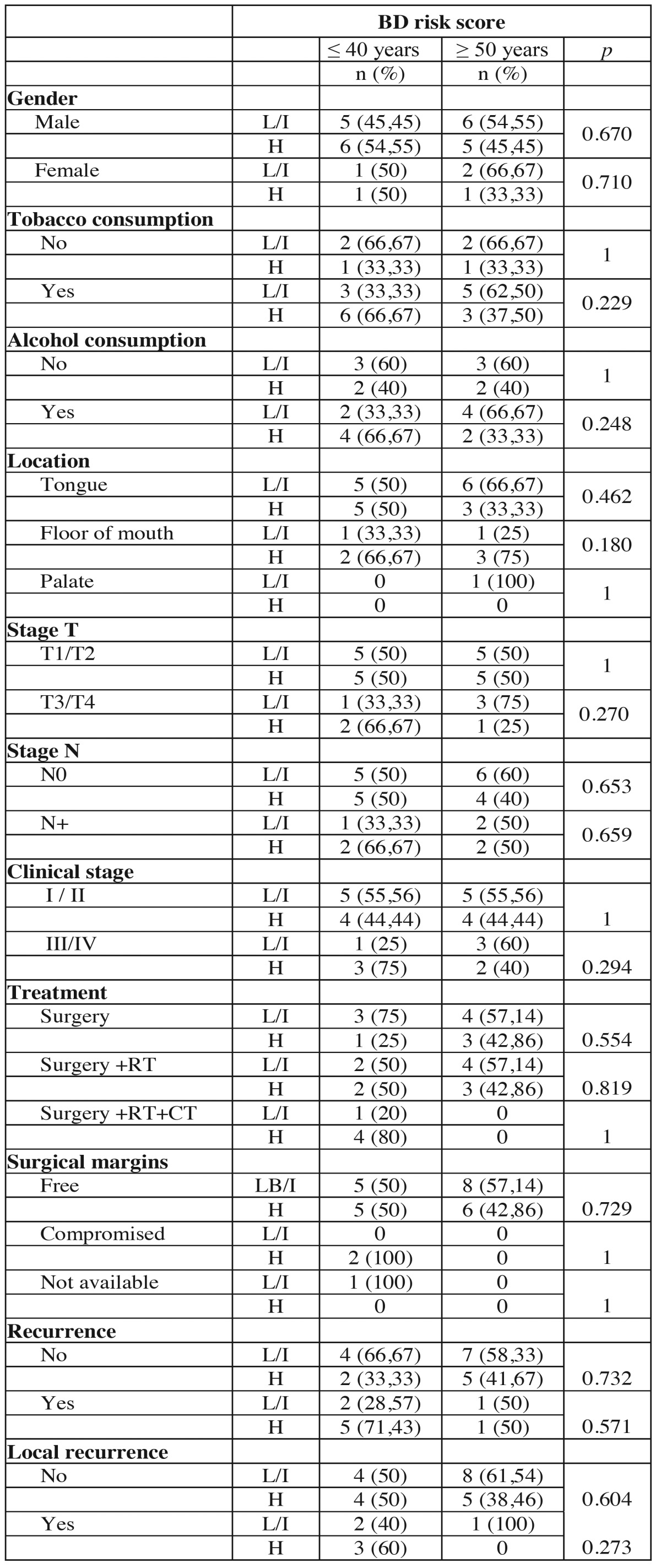
Association of clinical and demographic characteristics of the tumors of young patients (≤ 40 years) and control patients (≥ 50 years) with histopathological classification according to the BD risk score.

**Table 7 Continue:**
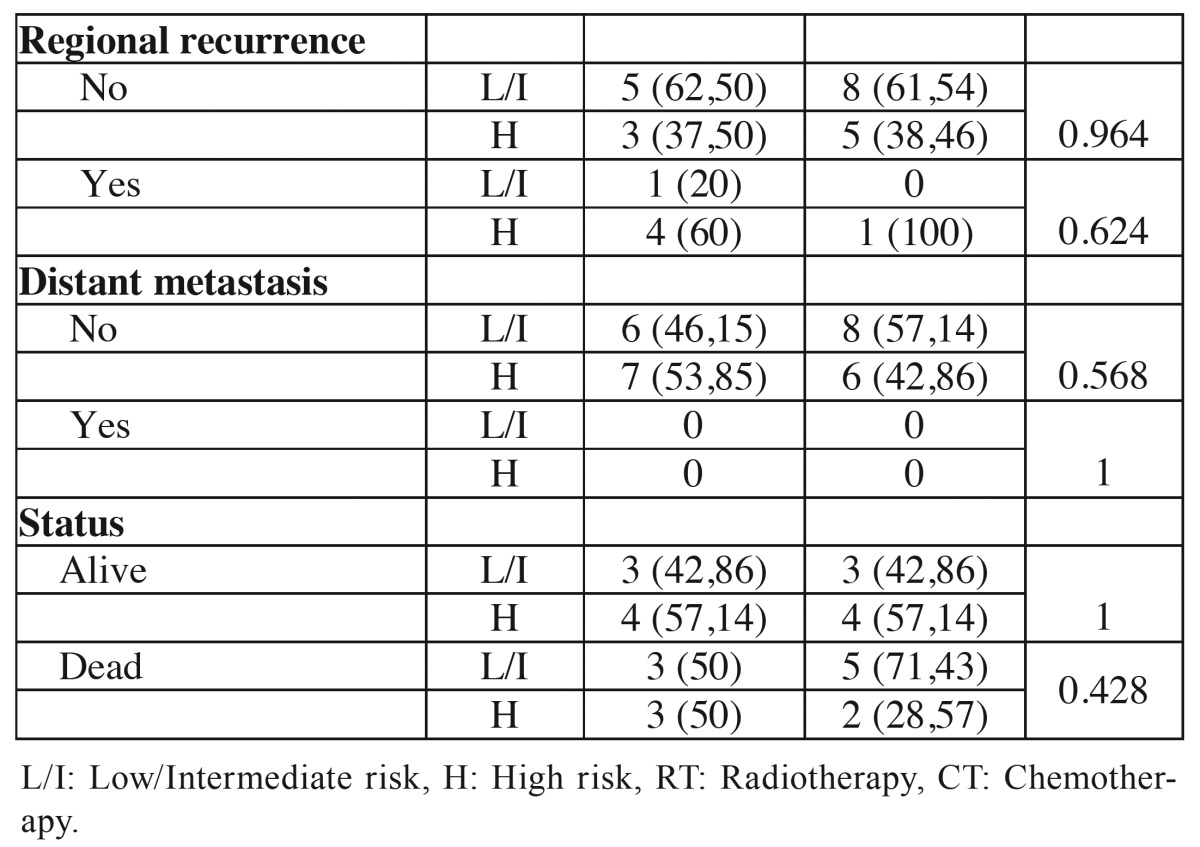
Association of clinical and demographic characteristics of the tumors of young patients (≤ 40 years) and control patients (≥ 50 years) with histopathological classification according to the BD risk score.

**Table 8:**
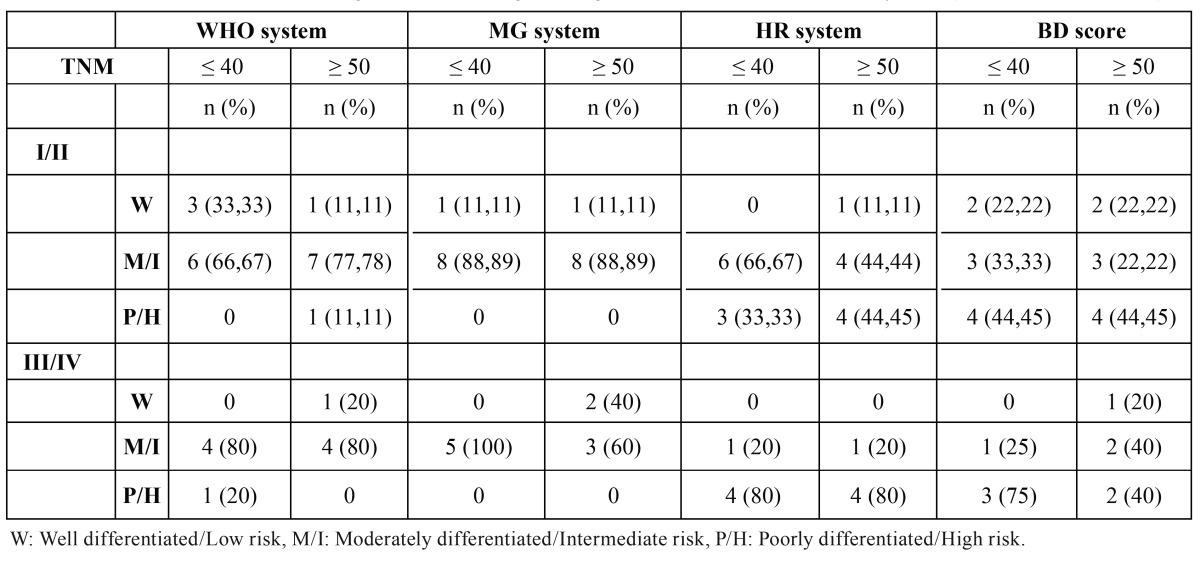
Distribution of tumors according to the clinical stage and degree of differentiation in the four systems (WHO, MG, HR and BD).

## Discussion

In Brazil, oral carcinoma is among the 10 most incidents cancers and it is estimated approximately 15,000 new cases for the year 2014. Among young patients, the incidence is considered low and retrospective analyzes are rarely higher rates to 6% of this tumor in this population. However, in recent years, it has been seen an increased incidence of oral carcinoma among patients younger than 40 years old (5,15-17). Hirota *et al.* (4) in a retrospective study conducted in Brazil between 1994 and 2004, observed incidence of SCC in young patients of 10.7%.

The prevalence of oral carcinoma is higher in male patients with variable ratios. In the study of Udeabor *et al.* (6), the ratio men:women was 3.8: 1. However, other studies showed lower ratios, being 1.6: 1 (4,5). In the current study the ratio was 6: 1, which is higher than other studies. There is no consensus in the literature about demographic characteristics, lifestyle, etiology, prognosis and results in young patients with oral carcinoma. In this study, it was found that the majority of patients were male (85.71%), consumed tobacco (71.43%) and alcohol (50%) and often the predominant location of the tumors was tongue (78.57%), similar data to the control group.

The fact that even when young patients have the risk factors of tobacco and alcohol, it has shorter period of time to induce carcinogenesis when compared to older patients; allowing new research about other etiologic factors responsible for the development of SCC in young individuals, such as genetic abnormalities and viral infections (4-6,8,18,19).

Furthermore, it has been suggested that oral carcinoma in young individuals could be related to the possibility of this tumor being a different type of cancer with apparently more aggressive biological behaviour (15,20). However, there is no consensus in the literature on this subject. Some studies found no significant differences between groups with different age groups regarding biological behavior of tumors (18,21).

In this study, the majority of young patients (64.28%) were diagnosed at an early stage (I and II), different than the study Benevenuto *et al.* (18) in which 67% of oral SCC in young patients were diagnosed in stages III and IV. The higher proportion of young patients with advanced tumors can be explained by delayed diagnosis, as also occurs in older patients (16) or by a possible more aggressive tumor behavior in this group of patient (8).

The parameters used to plan the treatment of patients with SCC are mainly based on clinical staging of the disease. The main form of treatment of oral carcinoma is surgery, which is frequently combined with other modalities such as radiotherapy and chemotherapy particularly in more advanced cases (22). Montero *et al.* (10), in a systematic review, from 2007 to 2012 on SCC features in young patients, noted that the evaluated studies showed a predominance of surgery for treatment of young patients, followed by combination surgery + radiotherapy. According to the authors, the association of chemotherapy would be a suitable option for more advanced tumors, with margins showing neoplastic infiltration. They also noted that the treatment used to pump in young patients is similar to that used in older individuals. However, according to the analyzed studies, the young patients are often subjected to combination treatments, regardless of the stage of the disease, because some authors have reported that the SCC’s behavior is more aggressive in this group. In the current study, it was also observed that young patients received a higher association of treatment modalities than the control group, being the difference statistically significant (*p* = 0.022).

Affected surgical margins, according Binahmed *et al.* (23) may be associated with local recurrence and poorer survival rates. On the other hand, Brandwein-Gensler *et al.* (13) in their study, found that the histological grading is more important that the assessment of surgical margins in determining the prognosis. In the present study, it was observed that only 2 young patients presented compromised surgical margins and all patients of the control group had free surgical margins, but the difference was not significant.

One of the major clinical prognostic indicators is the nodal status, so that survival can decrease by 20% when regional metastases are present (24). In this study, neck dissection was performed in 10 (71.42%) young patients and in 7 (50%) control patients and histopathological confirmation of lymph node involvement was seen in only 2 patients of each group and the difference was not significant.

Regarding recurrence and metastasis, Siriwardena *et al.* (20) observed a higher recurrence rate in young (39%) than in older patients (30%). In the review by Montero *et al.* (10) it was observed a controversy between analyzed studies with regard recurrence rates of oral carcinoma in young patients. In the current study there was a higher rate of local recurrence and regional metastasis among young people and the difference was statistically significant (*p* = 0.018).

An important feature of causal effects of age on survival are the comorbidities in other systems, which are more common in older patients and demonstrate a significant impact on the prognosis (25). In addition, overall survival rates seem to be more favorable in patients with no history of risk factors compared to those who use tobacco and alcohol, regardless of age (10). That is, a longer period associated with these diseases could lead to reduced patient survival. The study of Monsjou *et al.* (1) also found that younger patients had better OS rate, possibly due to the influence of comorbidities associated with old age. On the other hand, no significant difference in DFS rates among the young and elderly patients was noted. In the current study it was observed that young patients had DFS rate in 5 years significantly lower (37.68%) than the control group (77.78%), suggesting greater aggressiveness of tumors in the first group. However, in 10 years, this rate has remained in the young group and decreased in the control (18.84%). Regarding the OS rate, the difference between groups in 5 and 10 years was not significant.

The evaluation of prognostic factors of oral SCC has been widely studied in order to achieve more effective therapeutic strategies. Considering the poor prognosis of oral SCCs, as well as clinical studies, several histopathological grading systems have been developed trying to explain the differences in biological behavior of tumors with similar clinical characteristics (9). However, none of these systems is universally accepted (26). The current study used the systems developed by WHO, Bryne *et al.*(12), Brandwein-Gensler *et al.* (13) and Almangush *et al.* (14).

Regarding the histopathological grading of tumors, several authors found a similarity between groups of young and elderly patients (6,15,17,18). On the contrary, Kaminagakura *et al.* (21) found a higher frequency of poorly differentiated tumors in young patients compared to older.

Although the WHO system for histopathological classification of SCC is largely employed, their use as prognostic tool has been criticized. The main criticism of this system refers to their subjectivity, in the absence of important features related to tumorigenesis, such as pattern of invasion and, more importantly, the poor correlation with the results and responses to treatment (12). In the current study the WHO grading system in both groups ranked 13 tumors (92.85%) as well or moderately differentiated and 1 (7.15%) as poorly differentiated and the difference between them was not statistically significant.

Since it was described as an applicable grading system in biopsies, the MG system (12) has been used for prognostic analysis, but the results of the studies are controversial (9,24) which can be explained by the subjectivity attributed to some of their parameters resulting in high variability among examiners (27). In this study, the majority of tumors in both groups were classified as intermediate risk and again no difference was found between the groups.

The HR system (13) was proposed as a multiparameter system modified and updated with an important role in making decisions about the need for post-operative therapy and prognosis of patients with oral carcinoma. Although some studies, such as and Lindenblatt *et al.* (9) have confirmed their predictive value, other, more recently, such as Almangush *et al.* (14) and Rodrigues *et al.* (26) showed no correlation between this system and the epidemiological and clinical characteristics. According to Rodrigues *et al.* (26) none of the three parameters individually considered in the HR system as prognostic predictors has shown high reproducibility. In the current study, the HR grading system ranked 7 tumors of the youth group as low or intermediate risk and 7 as high prognostic risk. In the control group 6 tumor was classified as low or intermediate risk and 8 as high prognostic risk. It was observed statistical differences between the young and control groups regarding clinical stages III / IV, free margins and the absence of regional recurrence.

The BD risk score (14) is the latest proposal for histopathologic grading system of oral cancers. It’s two evaluation items, the depth of tumor invasion and tumor cell nests have been individually described as prognostic predictors for patients with oral carcinoma. The importance of these factors also have been highlighted in other studies. In the current study, there was no difference between the group of young patients and the control group patients compared to histological grading system for BD. Interestingly, in the HR and BD systems more tumors were classified as high risk prognosis than in the WHO and MG systems, suggesting that these systems can more accurately identify undifferentiated tumors than WHO and MG systems.

In summary, this study showed that no differences were observed in the histological grading in four used systems (WHO, MG, HR and BD) between young and control patients with tumors of similar location and clinical stage. However, even considering the limitation of the sample, it was noticed that younger patients had a higher rate of local recurrence, high rate of regional metastases and lower 5-year disease-free survival, despite of similar clinicopathological features and use of more therapeutic modalities.
